# Constitutional mismatch repair deficiency mimicking Lynch syndrome is associated with hypomorphic mismatch repair gene variants

**DOI:** 10.1038/s41698-024-00603-z

**Published:** 2024-05-24

**Authors:** Richard Gallon, Carlijn Brekelmans, Marie Martin, Vincent Bours, Esther Schamschula, Albert Amberger, Martine Muleris, Chrystelle Colas, Jeroen Dekervel, Gert De Hertogh, Jérôme Coupier, Orphal Colleye, Edith Sepulchre, John Burn, Hilde Brems, Eric Legius, Katharina Wimmer

**Affiliations:** 1 Translational and Clinical Research Institute, Faculty of Medical Sciences, Newcastle University, Newcastle upon Tyne, UK; 2 Centre for Human Genetics, University Hospital Leuven, Leuven, Belgium; 3 CHU, University of Liège, Liège, Belgium; 4 Institute of Human Genetics, Medical University of Innsbruck, Innsbruck, Austria; 5 Département de Génétique, AP-HP.Sorbonne Université, Hôpital Pitié-Salpêtrière, Paris, France; 6 Inserm UMRS_938, Sorbonne Université, Centre de Recherche Saint Antoine, Paris, France; 7 Département de Génétique, Institut Curie, Paris, France; 8 INSERM U830, Université de Paris, Paris, France; 9 Department of Digestive Oncology, University Hospital Leuven, Leuven, Belgium; 10 Department of Pathology, University Hospital Leuven, Leuven, Belgium; 11 Human Genetics, CHU Liège, Liège, Belgium; 12 Department of Pathology, CHU Liège, Liège, Belgium

**Keywords:** Cancer genetics, Diagnosis

## Abstract

Lynch syndrome (LS) and constitutional mismatch repair deficiency (CMMRD) are distinct cancer syndromes caused, respectively, by mono- and bi-allelic germline mismatch repair (MMR) variants. LS predisposes to mainly gastrointestinal and genitourinary cancers in adulthood. CMMRD predisposes to brain, haematological, and LS-spectrum cancers from childhood. Two suspected LS patients with first cancer diagnosis aged 27 or 38 years were found to be homozygous for an MMR (likely) pathogenic variant, *MSH6* c.3226C>T (p.(Arg1076Cys)), or variant of uncertain significance (VUS), *MLH1* c.306G>A (p.(Glu102=)). *MLH1* c.306G>A was shown to cause leaky exon 3 skipping. The apparent genotype-phenotype conflict was resolved by detection of constitutional microsatellite instability in both patients, a hallmark feature of CMMRD. A hypomorphic effect of these and other variants found in additional late onset CMMRD cases, identified by literature review, likely explains a LS-like phenotype. CMMRD testing in carriers of compound heterozygous or homozygous MMR VUS may find similar cases and novel hypomorphic variants. Individualised management of mono- and bi-allelic carriers of hypomorphic MMR variants is needed until we better characterise the associated phenotypes.

## Introduction

Loss of mismatch repair (MMR) function is found in several types of cancer, most commonly in gastrointestinal and genitourinary tumours^1^, and is an important biomarker for prognosis and therapeutic response, including resistance to chemotherapy and response to immune checkpoint inhibitors^2^. Two distinct cancer predisposition syndromes are associated with MMR deficient cancers, caused by germline pathogenic variants (PV) affecting one of four MMR genes, *MLH1* (MIM *120436), *MSH2* (MIM *609309), *MSH6* (MIM *600678), or *PMS2* (MIM *600259).

Lynch syndrome (LS; MIM #609310, #120435, #614350, #614337) is caused by mono-allelic germline MMR PVs and is one of the commonest cancer predisposition syndromes, with an estimated one in 279 of the general population carrying a germline MMR PV^3^. LS cancers are predominantly found in the gastrointestinal and genitourinary tracts, most frequently colorectal cancer (CRC) and endometrial cancer (EC). The typical age of disease onset is 40-60 years depending on affected gene, but cancer penetrance is incomplete^4^.

Constitutional mismatch repair deficiency (CMMRD; MIM #276300, #619096, #619097, #619101) is caused by bi-allelic germline MMR PVs and is much rarer than LS, with an estimated birth incidence of one in a million^5^. CMMRD typically presents with haematological, brain, and intestinal cancers in childhood or adolescence, with a median age of onset <10 years. CMMRD patients are highly likely to develop multiple malignancies^6^. CMMRD is also associated with several distinctive non-neoplastic features, most commonly café au lait maculae, other skin pigmentation alterations, and multiple developmental venous anomalies^6,7^. CMMRD patients may have an LS family history. These features are included in indication criteria for CMMRD testing in paediatric and adolescent cancer patients^6^.

Whilst LS and CMMRD patients both develop MMR deficient tumours that may share common treatment modalities, the difference in tumour spectra and age of onset require very different long term management. LS carriers are recommended 1-5 yearly gastrointestinal and gynaecological surveillance from age 20 years or older, depending on which MMR gene is affected and the specific guidelines^8–12^. In contrast, individuals with CMMRD are recommended annual gastrointestinal surveillance from as early as 6 years, annual brain magnetic resonance imaging (MRI) from initial diagnosis or at latest aged 2 years, and annual clinical examination, among other interventions^13–17^. Genetic counselling and psychological impact will also be considerably different between CMMRD and LS.

Here, we describe two cases of late onset CMMRD where the first cancer diagnosis was either an ovarian cancer or EC at ages 27 years and 38 years respectively, mimicking LS and causing diagnostic uncertainty. Observations from these and additional cases identified in the literature have significant implications for diagnosis and personalised management of CMMRD and LS.

## Results

### Case 1

The Case 1 patient had a first cancer diagnosis at the age of 27 years following clinical investigation due to an intra-abdominal mass. Biopsy of the tumour showed a clear cell carcinoma of the right ovary (stage IIIa). The patient was treated with cisplatinum and cyclophosphamide followed by debulking surgery. Incidentally, an EC was found (stage Ia). Four years later, an intra-abdominal nodulus in the region of the recto-sigmoid was seen on an abdominal computerised tomography scan, representing a relapse of the ovarian carcinoma. The patient was treated with cisplatinum and paclitaxel followed by resection of the nodulus and the recto-sigmoid. After surgery, the patient was treated with doxorubicin followed by ifosfamide. One year later, an autologous stem cell transplantation was performed after panabdominal irradiation. Aged 39 years, the patient had part of the small intestine resected due to intestinal sub-obstruction caused by adhesions. During follow up endoscopies, duodenal adenomatous polyps were found and resected. At the age of 43 years, a colonic adenocarcinoma (pT3N0M0) at the hepatic flexure was diagnosed, following iron deficiency anaemia and blood in the stools, and treated by a right-sided hemicolectomy and 5-fluorouracil. Immunohistochemistry (IHC) showed loss of MSH6 expression in tumour and non-neoplastic cells but was considered technically unreliable. The tumour was microsatellite instability (MSI)-high.

**Fig. 1:**
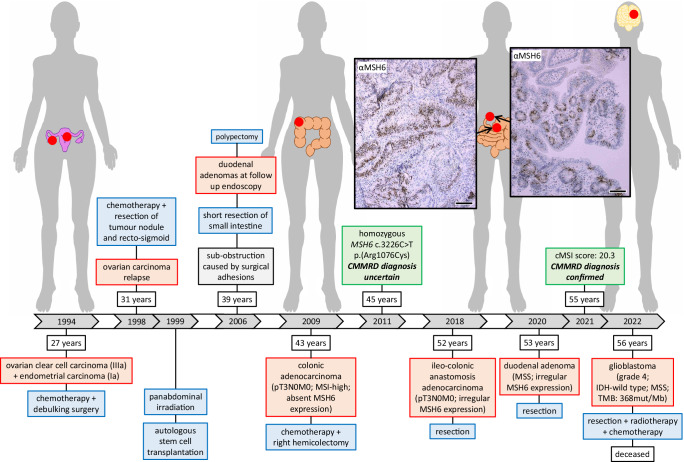
Clinical course of Case 1. The patient’s timeline of clinical presentation (red boxes), diagnosis (green boxes), and management (blue boxes). The scale bar in the bottom right corner of each immunohistochemistry image represents 100 μm. Immunohistochemistry controls to demonstrate specificity of staining are available in Supplementary Fig. S1.

**Fig. 2:**
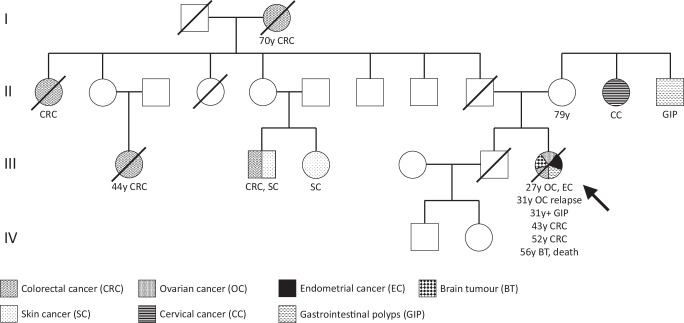
Case 1 patient’s pedigree. The circles denote females and the squares denote males. Filled symbols indicate the individual had a tumour, with the age at tumour diagnosis being given in years (y) if known. Deceased individuals are indicated with a strike-through. The patient is denoted by the arrow.

The patient’s clinical history (Fig. 1) did not indicate testing for CMMRD and no CMMRD-related non-neoplastic features were noted, but the phenotype and pedigree, which included several CRC diagnoses on the paternal side (Fig. 2), were suggestive of LS. Surprisingly, germline genetic testing of the patient identified a homozygous *MSH6* missense variant: c.3226C>T (p.(Arg1076Cys)). *MSH6* c.3226C>T is reported in the gnomAD v2.1.1 database with an allele frequency of 0.0079% in the European (non-Finnish) population (the patient’s ancestry) and was classified as a variant of uncertain significance (VUS) by the International Society for Gastrointestinal Hereditary Tumours (InSiGHT) at the time of detection in 2011, making the diagnosis uncertain. The patient was managed according to a clinical diagnosis of LS aged 45 years. In the following years, duodenal and colonic polyps were resected at each surveillance endoscopy. At the age of 52 years, the patient presented with abdominal pain and blood in the stools and a new colonic adenocarcinoma was diagnosed at the ileo-colonic anastomosis (pT3N0M0). The patient was treated surgically. IHC showed MSH6 expression in the tumour cells but with an irregular pattern. Nearly two years later, aged 53 years, the patient had a tubular adenoma of the duodenum resected. IHC again showed irregular expression of MSH6 in dysplastic cells and MSI analysis found the adenoma to be MSS (Fig. 1, see Supplementary Figure S1 for IHC negative controls).

Around the time of these tumour diagnoses, in 2019, *MSH6* c.3226C>T was reclassified as (likely) pathogenic by InSiGHT following its detection in trans with truncating *MSH6* variants in patients with a CMMRD phenotype^18–22^, and as a heterozygous variant in patients with an LS phenotype^23–26^ (Supplementary Table S1). However, there were no cancer diagnoses in carriers of *MSH6* c.3226C>T related to the compound heterozygous CMMRD patients^18–22^. Furthermore, the incidental finding of this variant in patients without cancer, with non-LS spectrum cancer, or with MMR proficient CRC by our local genetics services did not support its pathogenicity (Supplementary Table S1). These considerations along with the Case 1 patient’s LS phenotype and variable tumour MMR status despite homozygosity for *MSH6* c.3226C>T meant the diagnosis remained uncertain. A highly accurate assay for constitutional MSI (cMSI) in peripheral blood leukocytes (PBLs), a hallmark feature of CMMRD, became available also at this time^27^, providing an ancillary test of MMR function in non-neoplastic tissues to resolve uncertain genetic diagnoses^6,28^. The patient had a cMSI score of 20.3, which is consistent with the previously published cMSI score range of *MSH6*-associated CMMRD patients (20.9-152.7) and is much greater than the cMSI scores of LS carriers (0.0-11.3) or controls (0.0-3.6) (Supplementary Figure S2). As such, cMSI analysis confirmed a CMMRD diagnosis and corroborated classification of *MSH6* c.3226C>T as (likely) pathogenic.

Shortly after, aged 56 years, the patient was diagnosed with a brain tumour following an epileptic attack. The tumour was located in the left frontal lobe and classified as a high grade glioblastoma (grade 4). It was IDH wild type, MSS, and ultra-hypermutated with 368 mutations per megabase. CMMRD high grade brain tumours are often (ultra-)hypermutated due to somatic *POLE* or *POLD1* exonuclease domain variants that impair polymerase proofreading, leading to a complete loss of replication error repair^29–31^. Analysis of COSMIC single base substitution (SBS) signatures found that 20.7% (*n* = 147) of the somatic single nucleotide variants (SNV) are attributable to signature SBS10b, in agreement with a Pol ε proofreading defect. The tumour also had four somatic *POLE* missense variants (Supplementary Table S2). Two of these were notable. *POLE* c.1288G>T (p.(Ala430Ser)) is located in the Exo II motif of the exonuclease domain and has been reported once as a germline variant in a patient suspected of hereditary CRC in ClinVar. Whilst this alanine residue is highly conserved in higher eukaryotes, it is replaced by a serine in yeast and was predicted to be benign by AlphaMissense (Supplementary Table S2), suggesting the p.(Ala430Ser) variant may not be the cause of ultra-hypermutation in the patient’s tumour. *POLE* c.2385G>T (p.(Lys795Asn)) is located in the polymerase catalytic domain and alters a highly conserved residue. It has not been reported previously but was classified as ambiguous pathogenicity by AlphaMissense with a score close to the ambiguous-pathogenic classification threshold (Supplementary Table S2). Polymerase catalytic domain variants have been associated with somatic hypermutation in cancer^30^ and have recently been confirmed as drivers of mutagenesis^32^, suggesting the p.(Lys795Asn) variant may be the cause of ultra-hypermutation. Germline *POLE* and *POLD1* variants cause polymerase proofreading-associated polyposis (PPAP) and have been associated with both LS-like and CMMRD-like phenotypes^33–35^. Therefore, to exclude a germline origin for the *POLE* variants detected in the glioblastoma, the Case 1 patient’s PBL genomic DNA was sequenced but no *POLE* or *POLD1* variants were detected. The mutational signature analysis of the brain tumour also identified signatures SBS36 (38.9% of SNV) and SBS42 (40.3% of SNV). SBS36 is associated with deficiency of base excision repair due to loss of *MUTYH* function, but no somatic or germline variants in *MUTYH* had been found. Hence, the cause of this signature remains unknown, though it is possible a variant in *MUTYH* or a different gene involved in the base excision repair pathway was not detected. SBS42 is associated with haloalkane exposure. The patient was treated by surgery, radiotherapy and temozolomide for the glioblastoma, and died 6 months later (Fig. 1).

### Case 2

The Case 2 patient had a first cancer diagnosis at the age of 38 years following hysteroscopic investigation for menometrorrhagia despite contraceptive use. An endometrial polyp was removed by hysteroscopic polypectomy and found to be a grade 2 endometrioid adenocarcinoma (pT1aN0M0). The tumour retained expression of all four MMR proteins by IHC but was MSI-high, indicating MMR deficiency. A positron emission tomography scan was negative for additional tumours. Subsequently, the patient had a hysterectomy with pelvic sentinel lymph node mapping. Histology revealed no residual tumour and the lymph nodes were also negative. No additional treatment was necessary beyond routine follow up. A thoracoabdominal scan one year later was negative for signs of neoplasia.

**Fig. 3:**
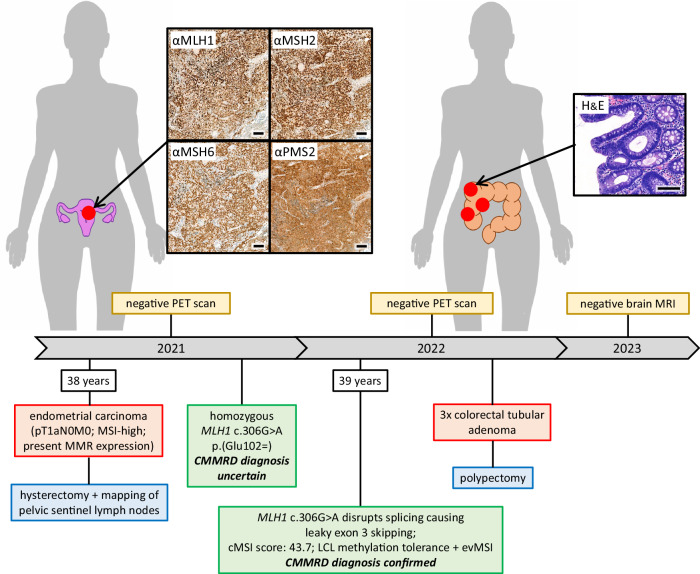
Clinical course of Case 2. The patient’s timeline of clinical presentation (red boxes), diagnosis (green boxes), and management (blue and yellow boxes). The scale bar in the bottom right corner of each immunohistochemistry or haematoxylin and eosin (H&E) image represents 100 μm. Immunohistochemistry controls to demonstrate specificity of staining are available in Supplementary Fig. S1.

**Fig. 4:**
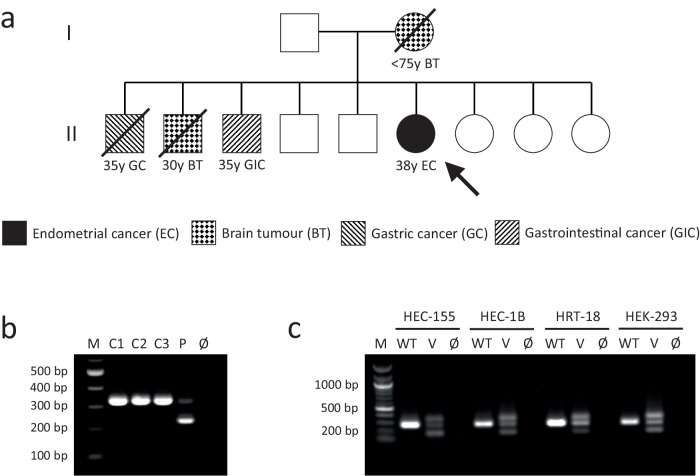
Case 2 patient’s pedigree and confirmation of a splice effect of *MLH1* c.306G>A. **a** The patient’s pedigree. The circles denote females and the squares denote males. Filled symbols indicate the individual had a tumour, with the age at tumour diagnosis being given in years (y) if known. Deceased individuals are indicated with a strike-through. The patient is denoted by the arrow. **b** Transcript analysis by PCR amplification of cDNA generated from RNA extracted from PHA-stimulated and puromycin-treated short-term cultures of peripheral blood leukocytes. The PCR primers used are located in *MLH1* exon 1 and exon 4. The expected amplicon sizes are 333 bp from full-length *MLH1* transcript and 234 bp from *MLH1* transcript lacking exon 3 (99 bp). C1-C3: control (*MLH1* wild type) leukocyte cDNA. P: patient (*MLH1* c.306G>A homozygous) leukocyte cDNA. Ø: negative-template control. **c** Analysis of minigene transcripts by PCR amplification of cDNA from RTB minigene-transfected cell lines (HEC-155, HEC-1B, HRT-18, and HEK-293) using primers located in RTB minigene exon 1 and exon 4 (Supplementary Figure S7). The expected amplicon sizes are 267 bp from RTB minigene transcript containing *MLH1* exon 3 and 168 bp from transcript lacking *MLH1* exon 3 (99 bp). WT: cDNA amplicons from cells transfected with RTB minigene containing wild type *MLH1* exon 3. V: cDNA amplicons from cells transfected with RTB minigene containing variant (c.306G>A) *MLH1* exon 3. Ø: negative-template control. Note: Amplification of cDNA from the RTB minigene containing variant c.306G>A *MLH1* exon 3 produces an unexpected third, larger amplicon that appears to be a heteroduplex of the other products based on Sanger sequencing data (Supplementary Fig. S5).

The patient’s phenotype (Fig. 3) did not indicate CMMRD testing and no other clinical features of CMMRD were identified, but LS was suspected. Pedigree data were available from first degree relatives only. Of note, three of the patient’s eight siblings were diagnosed with cancer in their thirties: One had a gastric cancer aged 35 years, a second a brain tumour aged 30 years, and a third a gastrointestinal tract cancer (undisclosed location) aged 35 years (Fig. 4a).

Germline genetic testing identified a homozygous synonymous variant altering the last nucleotide of *MLH1* exon 3, c.306G>A (p.(Glu102=)). *MLH1* c.306G>A is reported in the gnomAD v2.1.1 database with an allele frequency of 0.0098% in the South Asian population (the patient’s ancestry). It is currently classified as a VUS by InSiGHT but splice site prediction algorithms suggested an effect on the *MLH1* intron 3 splice donor site. A similar effect was predicted for c.306G>C, which has previously been shown to cause exon 3 skipping^36^ (Supplementary Figure S3). Indeed, transcript analysis of the patient’s PBLs showed a shorter cDNA amplicon compared to controls, with a size difference of approximately 99 bp, consistent with skipping of *MLH1* exon 3. A faint amplicon of the expected size from amplification of full-length transcript suggested that exon 3 is retained in a small proportion of the patient’s transcripts (Fig. 4b). Sanger sequencing of these amplicons confirmed exon 3 skipping in the majority of the patient’s transcripts but revealed additional unexpected sequences. Fragment length analysis showed these additional sequences originate from two amplicons not visible by gel electrophoresis, each 5nt shorter than the amplicons of full-length transcript or transcript lacking exon 3 (Supplementary Figure S4). These are likely generated from alternate splicing using cryptic splice sites found 5nt upstream of the intron 3 splice donor site in exon 3 and 5nt downstream of the intron 1 splice acceptor site in exon 2 (Supplementary Figure S3). Use of the cryptic splice donor site in exon 3 is also seen in controls and, hence, likely represents naturally occurring alternative splicing^37^. Fragment length analysis was also used to estimate that ~5% of the patient’s *MLH1* transcripts were of full-length sequence (Supplementary Figure S4).

The mis-splicing caused by *MLH1* c.306G>A in the patient’s PBLs was supported by a minigene assay. Skipping of exon 3 was observed in all four cell lines expressing the *MLH1* c.306G>A variant minigene but not the wild type minigene. Residual expression of full-length *MLH1* transcript was also observed from the variant minigene (Fig. 4c). The identities of the amplicons from minigene transcript analysis were confirmed by Sanger sequencing, which also supported alternate splicing using the cryptic splice donor site in exon 3 that is 5nt upstream of the natural splice site (Supplementary Figure S5). Taken together, these functional data show *MLH1* c.306G>A disrupts splicing, causing exon 3 skipping (r.208_306del) and in-frame loss of 33 amino acids (p.Lys70_Glu102del), though some normal splicing and, hence, residual expression of wild type protein (p.Glu102=) is retained. This justifies its reclassification as (likely) pathogenic, at least in the context of a recessive disorder, and, hence, a diagnosis of CMMRD in the patient. Samples from the patient’s siblings were not available for genetic testing.

As a final confirmation of the CMMRD diagnosis, ancillary tests assessing MMR function in normal tissues were used. The patient had a cMSI score of 43.7, which is consistent with previously published scores of *MLH1*-associated CMMRD patients (54.1-292.8) and is much greater than scores of LS carriers (0.0-11.3) or controls (0.0-3.6) (Supplementary Figure S2). Further ancillary tests were also positive for CMMRD: Germline MSI (gMSI) analysis of patient PBLs found instability in 2/3 markers, and patient-derived lymphoblastoid cells developed ex vivo MSI (evMSI) and were tolerant of methylating agents (Supplementary Figure S6). As for Case 1, PPAP was excluded by germline genetic testing of polymerase genes, which found no *POLE* or *POLD1* variants.

Following a CMMRD diagnosis, the patient had a screening colonoscopy and three colorectal adenomas with tubular histology and low grade dysplasia were found. The patient also had a brain MRI, which was negative for signs of a tumour (Fig. 3). Going forward, the patient will be managed according to CMMRD surveillance guidelines^13–15^, including annual clinical examination, annual ileocolonoscopy, annual brain MRI, and annual urine cytology.

### Additional late onset CMMRD cases

The literature was reviewed for additional genetically-confirmed CMMRD cases with a late onset of disease, defined here as patients who had a first cancer diagnosis at an age ≥25 years, and so would not fulfil C4CMMRD criteria to indicate CMMRD testing^6^. The present study and 6 additional publications^38–43^ have reported on 13 such cases. Among these, there were up to 27 distinct cancer diagnoses (excluding known relapses), including 19 intestinal tract cancers (18 of which were CRC), 5 genitourinary tract cancers (2 ECs, 1 ovarian cancer, 1 bladder cancer, and 1 prostate cancer), as well as 1 melanoma and 2 glioblastomas. There were no haematological malignancies diagnosed in these late onset CMMRD cases. Six patients were reported to have multiple intestinal polyps or adenomas. Two patients were reported to have café au lait maculae (Table 1).

**Table 1:** Late onset CMMRD patients with first cancer diagnosis at age ≥ 25 years

Publication	Patient ID	Gene (Transcript ID)	Variant 1	Variant 2	Cancer Diagnoses (age in years) - Molecular Pathology	Other Clinical Features (age in years)
Present study	Case 1	*MSH6* (NM_000197.3)	c.3226C>T p.(Arg1076Cys) missense	c.3226C>T p.(Arg1076Cys) missense	OC (27) EC (27) OC relapse (31) CRC (43) - absent MSH6 expression, MSI-high CRC (52) - irregular MSH6 expression GBM (56) - IDH-WT, MSS, TMB 368mut/Mb, *POLE* variants	Duodenal and colorectal adenomas and polyps (39 + ) Duodenal adenoma (53) - irregular MSH6 expression, MSS LS family history
	Case 2	*MLH1* (NM_000249.4)	c.306G>A ^a^ p.[(Lys70_Glu102del,Glu102=)] loss of 33 AA, silent	c.306G>A ^a^ p.[(Lys70_Glu102del,Glu102=)] loss of 33 AA, silent	EC (38) - present MMR expression, MSI-high	Three colonic adenomas (39) Three affected siblings with either GC (35), BT (30), or GIC (35)
Gardès et al. ^38^	P3	*MSH6* (NM_000197.3)	c.3226C>T p.(Arg1076Cys) missense	c.3226C>T p.(Arg1076Cys) missense	CRC (45) CRC (49)	Colorectal polyps (age not reported)
Lavoine et al. ^39^	11(15)	*PMS2* (NM_000535.7)	c.2531C>A p.(Pro844His) missense	c.2531C>A p.(Pro844His) missense	CRC (33) - MSI-high CRC (46)^**b**^	Two colonic adenomas (33) Sibling with CRC (20), GC (32), and GBM (32)
Li et al. ^40^ ^c^	III-3	*PMS2* (NM_000535.7)	c.2002A>G ^a^ p.[(Ile668*,Ile668Val)] truncating, missense	c.2002A>G ^a^ p.[(Ile668*,Ile668Val)] truncating, missense	CRC (26) - absent PMS2 expression DC (40)	Gastrointestinal polyps (age not reported) LS family history CALM Absent PMS2 expression in normal tissue
	NA	*PMS2* (NM_000535.7)	c.2002A>G ^a^ p.[(Ile668*,Ile668Val)] truncating, missense	c.2002A>G ^a^ p.[(Ile668*,Ile668Val)] truncating, missense	CRC (38)	None reported
	NA	*PMS2* (NM_000535.7)	c.2002A>G ^a^ p.[(Ile668*,Ile668Val)] truncating, missense	c.2002A>G ^a^ p.[(Ile668*,Ile668Val)] truncating, missense	CRC (31)	None reported
	NA	*PMS2* (NM_000535.7)	c.2002A>G ^a^ p.[(Ile668*,Ile668Val)] truncating, missense	c.2002A>G ^a^ p.[(Ile668*,Ile668Val)] truncating, missense	CRC (31)	None reported
Shuen et al. ^41^	MMR108	*PMS2* (NM_000535.7)	c.2531C>A p.(Pro844His) missense	c.1261C>T p.(Arg421*) truncating	CRC (27) CRC (27)	Four colorectal adenomas (27) Absent PMS2 expression in normal tissue
	MMR120	*MSH6* (NM_000197.3)	c.3226C>T p.(Arg1076Cys) missense	c.1421_1422dupTG p.(Gln475Glyfs*7) truncating	GBM (27) GBM relapse (28)	None reported
	MMR189	*PMS2* (NM_000535.7)	c.137G>T p.(Ser46Ile) missense	c.137G>T p.(Ser46Ile) missense	CRC (28) CRC (32) M (45) BC (49) CRC (50) CRC (52) PrC (53)	Absent PMS2 expression in normal tissue
Xie et al. ^42^	III.1	*MSH6* (NM_000197.3)	c.3226C>T p.(Arg1076Cys) missense	c.3226C>T p.(Arg1076Cys) missense	CRC (32) - subclonal absent MSH6 expression, MSS, TMB-high CRC relapse (34)	Neurofibromas (unknown) Fourth degree consanguineous parents LS family history CALM
Bruekner et al. ^43^	NA	*MSH2* (NM_000251.3)	c.188T>A p.(Val63Glu) missense	c.188T>A p.(Val63Glu) missense	CRC (25)	Two siblings with CRC (17 and 26)

*AA* amino acid, *CALM* cafe au lait macule, *CMMRD* constitutional mismatch repair deficiency, *LS* Lynch syndrome, *MSI* microsatellite instability, *MSS* microsatellite stable, *TMB* tumour mutation burden, *BC* bladder cancer, *BT* brain tumour (unknown classification), *CRC* colorectal cancer, *DC* duodenal cancer, *EC* endometrial cancer, *GBM* glioblastoma (astrocytoma), *GC* gastric cancer, *GIC* gastrointestinal cancer (unknown location), *HGG* high grade glioma (including primitive neuroectodermal tumour), *IC* intestinal cancer (assumed jenunal or ileal), *M* melanoma, *OC* ovarian cancer, *PrC* prostate cancer.

^a^The variant causes a “leaky” splice effect.

^b^A rectal cancer diagnosed at age 46 years was previously unreported.

^c^ Also see Biswas et al.^45^ for functional analyses of *PMS2* c.2002A>G.

Among the 13 late onset CMMRD patients, there were only 8 unique MMR variants representing all four MMR genes, including 2 splice variants, 4 missense variants, and 2 truncating variants (Table 1; Supplementary Table S3). Notably, each late onset CMMRD patient had at least one allele affected by a missense or splice variant, and copy number or truncating variants represented only 2/26 (7.7%) of their alleles. This is significantly lower than the 51/112 (45.5%, *p* = 2.4 × 10^−4^) alleles containing copy number or truncating variants in the 56 CMMRD patients analysed by Gallon et al., who had a median age of first cancer diagnosis of 7 years^27^. Two late onset patients from the literature were homozygous for *MSH6* c.3226C>T (p.(Arg1076Cys)), the same genotype as in Case 1, and another was compound heterozygous for this variant. Two other patients were either homozygous or compound heterozygous for another missense variant *PMS2* c.2531C>A (p.(Pro844His)). Four patients shared a genotype of being homozygous for *PMS2* c.2002A>G (p.[(Ile668*, Ile668Val)]), a known hypomorphic splice variant that has residual expression of full-length protein similar to the splice effect of the *MLH1* c.306G>A variant found in Case 2.

## Discussion

The C4CMMRD indication criteria for CMMRD testing are applicable to cancer patients whose first tumour is at an age less than 18 or 25 years depending on tumour type^6^. Both cases reported here had no indication for CMMRD testing with first cancer diagnoses at 27 or 38 years. Both were initially investigated for LS. When genetic testing found no heterozygous MMR variants but instead revealed homozygous MMR variants, the LS phenotypes made the diagnoses uncertain. Assays of MMR function in non-neoplastic tissues and experiments to show the splice effect of *MLH1* c.306G>A confirmed a CMMRD diagnosis. There were 11 other cases of genetically-confirmed, late onset CMMRD in the literature, defined here as patients with first cancer diagnosis at an age ≥25 years (Table 1). Their tumour spectrum consisted mostly of intestinal and genitourinary cancers, with few brain tumours, and no haematological malignancies. Although not all were genetically-confirmed to have CMMRD, affected siblings of the total 13 late onset patients also had gastrointestinal and brain cancers but no haematological malignancies. Four late onset CMMRD cases were identified from a cohort of CMMRD patients homozygous for a Nunavik founder splice variant in *PMS2*, c.2002A>G (p.[(Ile668*, Ile668Val)]). Other *PMS2* c.2002A>G homozygotes with earlier disease onset indicative of CMMRD have been reported but, collectively, they have an attenuated phenotype that is less severe than individuals carrying biallelic *PMS2* truncating PVs. The tumour diagnoses reported in 13 *PMS2* c.2002A>G homozygotes include 15 gastrointestinal tract cancers, 2 brain tumours, and no haematological malignancies^40^. Therefore, the tumour spectrum of these late onset CMMRD patients, as well as their siblings or individuals sharing their genotype, is similar to LS.

The relatively late age of cancer onset and the LS-like tumour spectrum observed in these CMMRD patients is likely caused by specific, hypomorphic missense or splice variants. For example, mechanistic analyses of the variant found in the late onset CMMRD case of Bruekner et al. showed that MSH2 p.Val63Glu retains partial MMR activity by reducing but not preventing mismatch binding^43,44^. In Case 1 and the case reported by Xie et al. ^42^, analyses of tumour MSI and MMR protein expression gave variable results, suggesting the MSH6 p.Arg1076Cys variant protein is expressed and retains partial MMR function. Expression of MLH1 was retained in the normal and tumour tissues of the Case 2 patient despite homozygosity for the splice variant *MLH1* c.306G>A. The Nunavik Inuit founder variant *PMS2* c.2002A>G creates a novel 5′ splice site that leads to loss of the last 5 nucleotides of exon 11 (r.2002_2006del) and consequently a premature stop codon p.Ile668*, but, similar to *MLH1* c.306G>A, has residual expression of full length transcript, leading to functional PMS2 protein containing a missense variant (p.Ile668Val)^40,45^. It is also notable that Case 1 and Case 2 patients have the lowest cMSI scores for *MSH6*- and *MLH1*-associated CMMRD patients, respectively, and that *PMS2* c.2002A>G homozygotes have similarly low cMSI scores^27^ (Supplementary Figure S2), suggesting a less severe disruption of MMR. It has been hypothesised that the exceptional cancer incidence in CMMRD is associated with an increased mutation rate in their MMR deficient normal soma^46^. Therefore, partial MMR activity of missense variants and residual expression of functional protein from “leaky” splice variants in these late onset CMMRD cases may cause lower mutation rates when compared to other CMMRD patients and, consequently, fewer cells undergoing neoplastic transformation and slower tumour progression. Several of these variants recurred in the patients collated in Table 1 and fewer truncating or copy number variants were found compared to a previously published CMMRD cohort, supporting that specific hypomorphic variants may be causing a distinct clinical phenotype.

It is probable that other hypomorphic MMR variants with the potential to cause attenuated forms of CMMRD are currently considered VUS or benign, and that this phenotype may be clinically under-recognised. LS or CMMRD caused by hypomorphic variants should be considered when a cancer patient has a family history of LS-associated cancers and/or has been found to have germline mono- or bi-allelic MMR VUS, even if the tumour retains MMR protein expression or is MSS. In particular, a suspected LS patient who is homozygous or compound heterozygous for MMR VUS should have ancillary tests for CMMRD to clarify the diagnosis and variant classification. We identified three patients from the literature during our search for late onset CMMRD cases who had biallelic MMR variants and a severe LS phenotype, where one or both MMR variants are currently classified as VUS or benign by InSiGHT. Kets et al. described two siblings each with multiple LS cancers and adenomas starting in their 30 s who were compound heterozygous for an *MSH2* PV (c.1-?_1076+?del) and VUS (c.1A>G; p.Met1?)^47^. Plaschke et al. described a 31 year old CRC patient who was compound heterozygous for an *MSH6* VUS (c.2295C>G; p.(Cys765Trp)) and benign variant (c.2633T>C; p.(Val878Ala))^18^.

Current guidelines for the management of CMMRD recommend intensive surveillance starting in early childhood^13–15^. These surveillance recommendations are highly effective for brain and intestinal tract tumours, whilst little benefit was seen for haematological malignancies^16,17^. However, applying these guidelines to late onset CMMRD patients, assuming they have a distinct phenotype caused by hypomorphic variants, would give relatively low yields, particularly for brain tumours, given their delayed disease onset and LS-like tumour spectrum, whilst exposing young patients to often invasive and stressful interventions. Whilst LS management guidelines suggest gastrointestinal and genitourinary surveillance start as early as age 20-25 years^8–12^, these recommendations may be insufficient for late onset CMMRD patients given the earlier tumour onset and incidence of brain tumours observed in siblings and other patients sharing late onset-associated genotypes. For example, among *PMS2* c.2002A>G homozygotes, the reported age of first cancer diagnosis ranges from 3 to 38 years^40^ and the siblings homozygous for *PMS2* c.2531C>A (p.(Pro844His)) had 13 years difference in age at first cancer^39^ (Table 1). Genetic counselling and discussions of cancer risk also need to be considered carefully given the attenuated but variable clinical presentation associated with these hypomorphic variants. Furthermore, the penetrance of hypomorphic MMR variants in the heterozygous state is uncertain and their classification as pathogenic may only be applicable in the context of CMMRD, though data from *MSH6* c.3226C>T carriers suggests heterozygous hypomorphic MMR variants can present as LS^23–26^. Therefore, management recommendations for late onset CMMRD patients and heterozygous family members currently need to be made on an individual basis until additional patients are identified to better describe this phenotype and its aetiology.

In conclusion, the two cases of late onset CMMRD described here and examples in the literature show that CMMRD can mimic LS and suggests that this less severe CMMRD phenotype is associated with hypomorphic MMR variants. Therefore, CMMRD is a differential diagnosis of LS and testing for CMMRD in suspected LS patients with compound heterozygous or homozygous MMR VUS is warranted irrespective of clinical presentation. Characterisation of additional patients with late onset CMMRD will help define whether this represents a distinct genotype and phenotype, which may have implications for patient management.

## Methods

### Patient data and ethics

Two late onset CMMRD patients, presented here as two case reports, were identified during the course of their clinical diagnosis, based on genetic testing and functional assays. Both patients gave written informed consent to publication of de-identified information relevant to the clinical presentation and management of their disease, including their genetic diagnosis, pathology, and treatment. These case reports have followed CARE guidelines (https://www.care-statement.org/). An additional 11 late onset CMMRD patients were identified through review of the peer-reviewed literature, and novel analyses in the present article used data published with patient consent or ethical approval as described by the original articles^38–43^. All aspects of this study were conducted according to the Declaration of Helsinki.

### Tumour analyses and germline genetic testing

Tumour histological and molecular analyses and germline genetic testing followed standard operating procedures of the clinical laboratories serving each case. Tumour MSI analyses used fragment length analysis of custom marker panels, a mix of mono- and di-nucleotide repeats for Case 1, and a mononucleotide repeat panel following Suraweera et al.^48^ and Buhard et al.^49^ for Case 2. Tumours were classified as MSI-high if >30%, MSI-low if ≤30%, and MSS if none of the markers showed instability^50^. Sequencing of the Case 1 glioblastoma used the TruSight Oncology 500 gene panel and NextSeq500 (Illumina, San Diego, CA) according to the manufacturer’s protocols. Sequence variants were filtered using the default settings for tumour mutation burden calculation provided by Illumina, with exclusion of germline variants using the gnomAD database (https://gnomad.broadinstitute.org/) and a minor allele frequency threshold of 0.1%. Mutational signatures were extracted from the filtered variants using SigProfilerMatrixGenerator^51^, SigProfilerExtractor^52^, and SigProfilerAssignment^53^ with default settings, and using the COSMIC mutational signatures data files v3.3 for genome GRCh37 (https://cancer.sanger.ac.uk/signatures/downloads/).

Germline variants are described in accordance with the Human Genome Variation Society guidelines (http://varnomen.hgvs.org/). Estimations of the population frequency of variants used gnomAD database v2.1.1 (https://gnomad.broadinstitute.org/). Nucleotide conservation scores generated by phyloP100way^54^ were obtained from Varsome (https://varsome.com/). Prediction of splice site effects of nucleotide variants used spliceAI scores^55^ obtained from SpliceAI Lookup with default settings (https://spliceailookup.broadinstitute.org/). Prediction of splice site effects in *MLH1* additionally used four splice site prediction programs available through the AlamutTM Visual Plus vs 1.7.2 software (Sophia Genetics SAS, Technopole Izarbel, France). Prediction of functional effects of missense variants used REVEL scores^56^ and AlphaMissense scores^57^ obtained from https://zenodo.org/records/7072866 and https://zenodo.org/records/8360242, respectively.

### Assays of mismatch repair function in constitutional tissue

Confirmation of CMMRD diagnoses used several tests of MMR function in non-neoplastic tissues. In all assays, control samples are derived from individuals without a cancer diagnosis who do not have a germline MMR PV. cMSI analysis of PBL genomic DNA followed the protocols described by Gallon et al. ^27^. In brief, molecular inversion probes were used to capture and amplify 32 mononucleotide repeat MSI markers from each sample. Amplicon libraries were sequenced at a target 5000x read depth using a MiSeq (Illumina). Using a custom bioinformatics pipeline, reference allele frequencies were extracted for the MSI markers and used to calculate a cMSI score by comparison to a reference distribution from 80 control PBL genomic DNA samples. Higher cMSI scores indicate higher MSI in constitutional tissues. gMSI analysis of PBL genomic DNA was performed according to Ingham et al. ^58^. PCR amplicons of 3 dinucleotide repeat MSI markers were analysed using an ABI PRISM 3500 Genetic Analyzer (Applied Biosystems, Vienna, Austria) and the PeakHeights software (https://dna-leeds.co.uk/peakheights/) to calculate gMSI peak height ratios. In-house classification thresholds, based on the mean + 3 standard deviations peak height ratio of 40 control samples, were used for each MSI marker as described previously^59^, with gMSI positivity being called when at least 2/3 MSI markers had a ratio above the threshold. Methylation tolerance and evMSI analysis of patient-derived lymphoblastoid cell lines followed the protocols of Bodo et al. ^60^. To assess methylation tolerance, patient- and control-derived lymphoblastoid cell lines (LCL) were exposed to N-Methyl-N’Nitro-N-Nitrosoguanidine (MNNG) at 1.25 µM, 2.5 µM, and 5 µM using 2 or 3 treatment pulses over 10 days. LCL survival was evaluated using the WST kit (Roche, Indianapolis, IN), Infinite F500 microplate reader (Tecan, Männedorf, Switzerland), and Xfluor4GENiosPro software (Tecan). Percentage of cell survival was calculated using the absorbance of MNNG-treated LCL relative to an untreated control. To assess evMSI, patient-derived LCL were cultured for 392 days after immortalisation. PCR amplicons of 3 mononucleotide repeat MSI markers from cultured LCLs were analysed using an ABI 3100 Genetic Analyzer 7 and Gene Mapper software v3.7 (Applied Biosystems). Allele distribution shifts indicating evMSI were detected by visual inspection. Control LCLs cultured up to 304 days have not shown evidence of evMSI^60^.

### Transcript and minigene analyses of *MLH1* splicing

PBL transcript analysis for Case 2 was performed by direct cDNA sequencing according to Etzler et al. ^61^. RNA extracted from short-term lymphocyte cultures (treated with puromycin prior to cell harvest to prevent nonsense-mediated decay) was reverse-transcribed using the SuperScript III Reverse Transcriptase (Invitrogen, Vienna, Austria) and random hexamers. cDNA was PCR amplified using primers annealing to *MLH1* exon 1 (5′-ATGTCGTTCGTGGCAGGG-3′) and exon 4 (5′-AGCCACATGGCTTATGCTGG-3′) to generate a 333 bp amplicon from full-length transcript. The exon 4 primer was FAM-labelled for fragment length analysis. PCR amplicons were separated and visualised using 2% agarose gel electrophoresis or using fragment length analysis by the ABI PRISM 3730 Genetic Analyzer and GeneMapper 4.1 software (Applied Biosystems). Sanger sequencing used PCR amplicons treated with ExoSAP-IT (GE Healthcare, Vienna, Austria), the Big Dye Terminator chemistry V1.1 (Applied Biosystems), and the ABI PRISM 3730 Genetic Analyzer (Applied Biosystems). Sanger sequencing data were analysed with the SeqNext version 26 software (JSI medical systems, Ettenheim, Germany) and the Sequence Scanner Software v1.0 (Applied Biosystems).

Minigene analysis for *MLH1* c.306G>A used the RTB hybrid-minigene^62^ provided by Dr Thomas Cooper (Baylor College of Medicine). Two minigene constructs, containing either the wild type or c.306G>A variant sequence of *MLH1* exon 3 (Supplementary Figure S7), were generated by the following protocol. *MLH1* exon 3, including flanking 670 bp of intron 2 and 212 bp of intron 3, was PCR amplified from control (wild type) or the patient’s (c.306G>A variant) peripheral blood leukocyte gDNA using primers targeting *MLH1* intron 2 (5’-GGCGGCGTCGACACTGCTAATTTTAAAGCTCTTCTCA-3’) and intron 3 (5’-GCCGCCGGTACCTATCAGAGGTCTCTGCAGGT-3’) that introduce *Sal*I and *Kpn*I restriction sites (underlined in the primer sequences) at the 5´ and 3´ends of the amplicons, respectively. Amplicons were digested with *Sal*I and *Kpn*I restriction enzymes and ligated into respective restriction sites located between exon 2 and exon 4 of the RTB minigene. Wild type and variant minigene constructs were selected by colony PCR and Sanger sequencing. The integrity of the final constructs was confirmed by Sanger sequencing of the plasmid maxi‐preparations that were used for transient transfection into human cell lines. The minigene constructs were transfected into EC cell lines HEC-115 and HEC-1B, CRC cell line HRT-18, and embryonic kidney cell line HEK-293 (grown to approximately 60% confluence) using Turbofect reagent (Thermo Scientific, Waltham, MA) following the manufacturer’s protocol. Messenger RNA was extracted 48 hr after transfection using RNeasy Mini Kit (Qiagen, Limburg, The Netherlands) and reverse transcribed using random hexamers and Maxima H Minus First Strand cDNA Synthesis Kit (Thermo Scientific). PCR amplification of cDNA template used primers located in exon 1 (5′-GTGTGCACCTCCAAGCTC-3′) and exon 4 (5’-TAGAAAGTTGCATGGCTGG-3′) of the RTB minigene. PCR amplicons were separated and visualised using 2% agarose gel electrophoresis. The individual bands of the PCR amplicons were excised from the gel, purified using peqGOLD Gel Extraction kit (Peqlab Biotechnologie GmbH, Erlangen, Germany), and Sanger sequenced using the same primer pair, ABI PRISM 3730 genetic analyser (Applied Biosystems), and software as described above.

### Statistical analysis

All data analyses used R version 4.2.2 (https://cran.r-project.org/). Fisher’s exact test was used to test the statistical significance of the difference in frequency of MMR alleles containing either copy number or truncating variants in the 13 late onset CMMRD cases of Table 1 compared to a previously published cohort of 56 CMMRD patients^27^.

### Reporting summary

Further information on research design is available in the Nature Research Reporting Summary linked to this article.

### Supplementary information

Supplementary Information

Reporting summary

## Data Availability

For access to the study data, please contact the corresponding authors Dr Richard Gallon (richard.gallon@newcastle.ac.uk) and Associate Professor Katharina Wimmer (katharina.wimmer@i-med.ac.at).

## Code availability

Code availability is not applicable to this article as no custom code or mathematical algorithm were central to the conclusions of the current study.
