# Supplementary material for: Constitutional mismatch repair deficiency mimicking Lynch syndrome is associated with hypomorphic mismatch repair gene variants
Source: NPJ Precis Oncol. 2024 May 24;8:119. doi: 10.1038/s41698-024-00603-z (PMC11126593; doi:10.1038/s41698-024-00603-z)
Supplement: Supplementary file 1 — Supplementary Information [file 41698_2024_603_MOESM1_ESM.pdf]

Supplementary Figure S1: Immunohistochemistry controls.

a

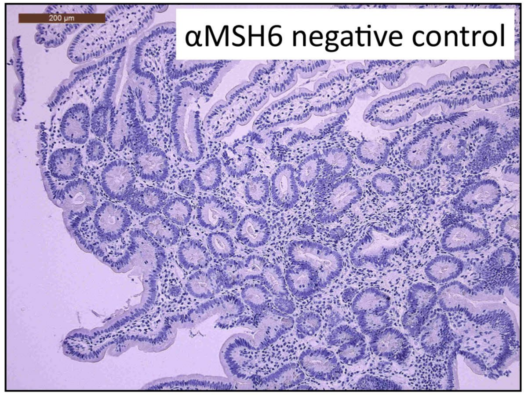

a Primary antibody negative control for αMSH6 immunohistochemical staining of the duodenal adenoma of Case 1. The scale bar in the top left corner represents 200µm. **b** Isotype controls for αMLH1, αMSH2, αMSH6, and αPMS2 immunohistochemical staining of the endometrial cancer of Case 2. The scale bar in the top left corner of each image represents 100µm.

b

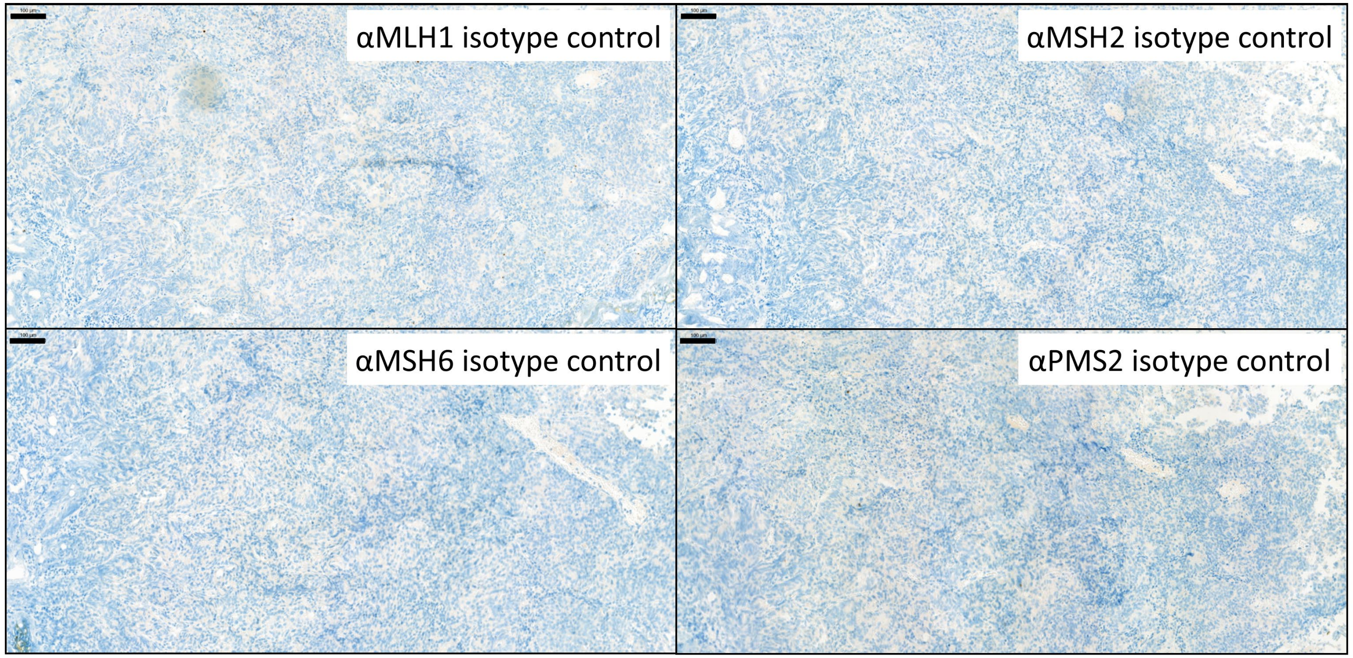

Supplementary Figure S2: Constitutional microsatellite instability analysis.

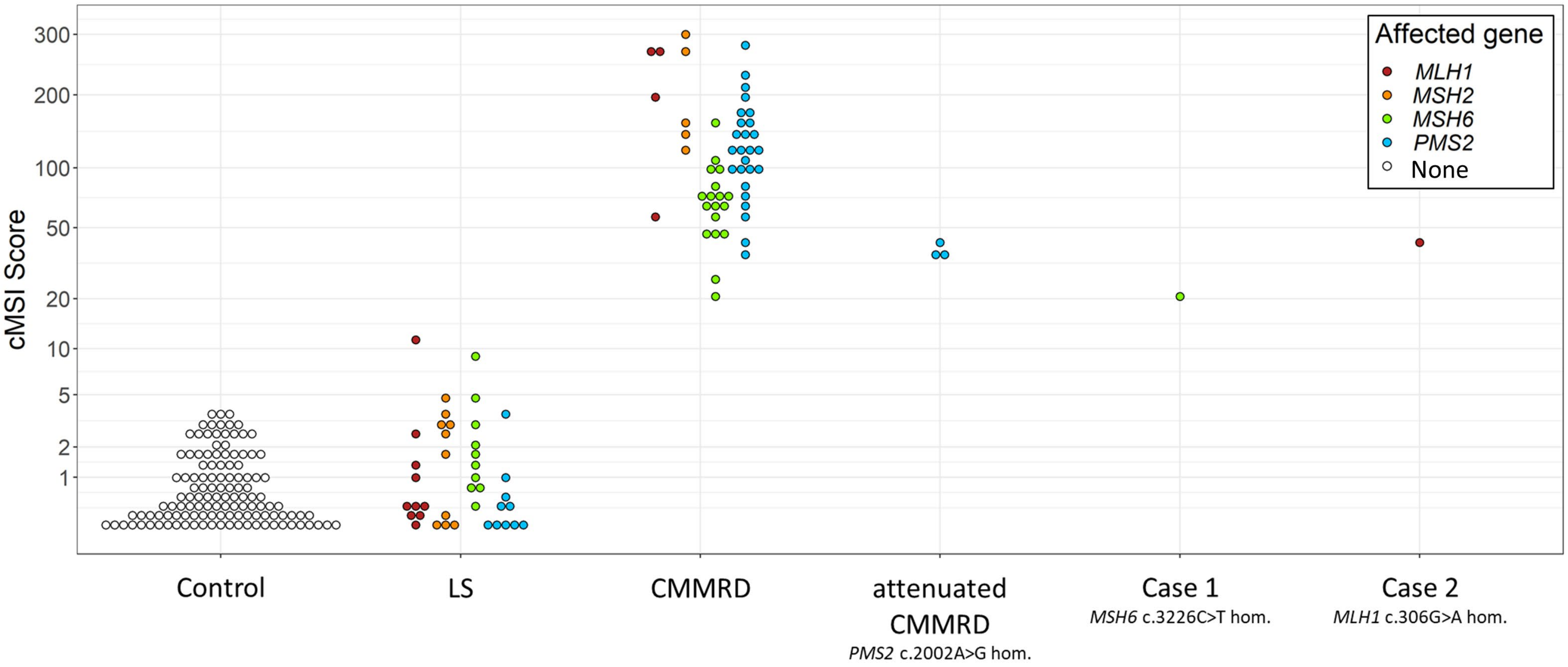

Constitutional microsatellite instability (cMSI) scores (Gallon et al., 2023; PMID: 36586540) of case patients compared to control, Lynch syndrome (LS), and constitutional mismatch repair deficiency (CMMRD) samples. Control, LS, and CMMRD cMSI scores are taken from Gallon et al. 2023. The y-axis is scaled based on a logit transformation. hom. = homozygous.

Supplementary Figure S3: The effect of *MLH1* c.306G variants on splice site prediction scores and the identification of potential cryptic splice sites for alternate splicing of *MLH1* transcripts.

a *MLH1* intron 3 splice donor site

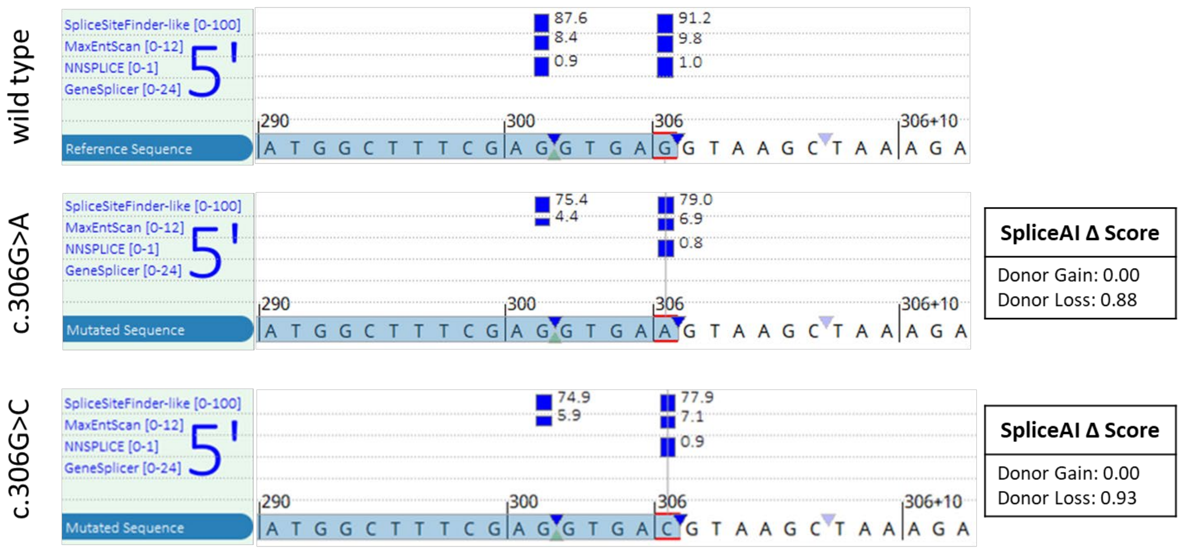

b *MLH1* intron 1 splice acceptor site

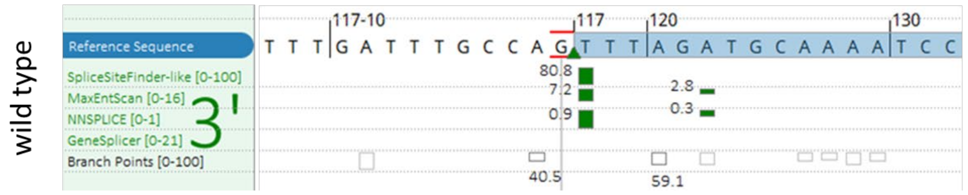

a Prediction of splice sites in wild type (top), c.306G>A variant (middle), and c.306G>C variant (bottom) sequences of the *MLH1* intron 3 splice donor site using four splice site prediction programs available through the Alamut^{TM} Visual Plus vs 1.7.2 software (Sophia Genetics SAS, Technopole Izarbel, France). The two variant sequences similarly reduce the splice prediction scores compared to the wild type sequence. Note, there is a cryptic splice donor site 5nt upstream of the natural splice site with only slightly lower prediction scores. This cryptic splice site is present in the wild type and variant sequences and could be responsible for the 328bp transcript amplicons (5nt shorter than expected) observed by fragment length analysis in control and Case 2 patient peripheral blood leukocytes (see Supplementary Figure S4). SpliceAI Δ Score values represent the maximum difference in predicted splice site score using SpliceAI (Jaganathan et al. 2019, PMID: 30661751) between variant and wild type sequences. Both variants show an almost certain reduction in splice donor site activity according to SpliceAI (Δ Score >0.8). **b** Prediction of splice sites in wild type sequence of the *MLH1* intron 1 splice acceptor site using the Alamut^{TM} Visual Plus vs 1.7.2 software. There is a cryptic splice acceptor site 5nt downstream of the natural splice site with low prediction scores. This cryptic splice site could be responsible for the 229bp transcript amplicon (5nt shorter than expected when *MLH1* exon 3 is skipped) observed by fragment length analysis in Case 2 patient peripheral blood leukocytes (see Supplementary Figure S4).

Supplementary Figure S4: Fragment length analysis of *MLH1* transcript amplicons generated from peripheral blood leukocyte cDNA.

a

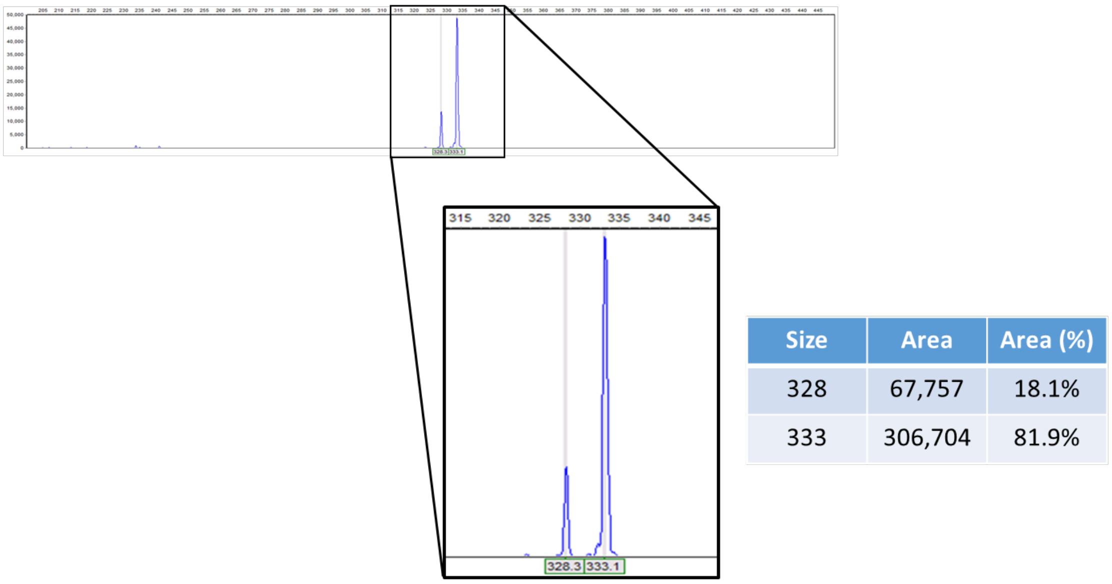

Size	Area	Area (%)
328	67,757	18.1%
333	306,704	81.9%

b

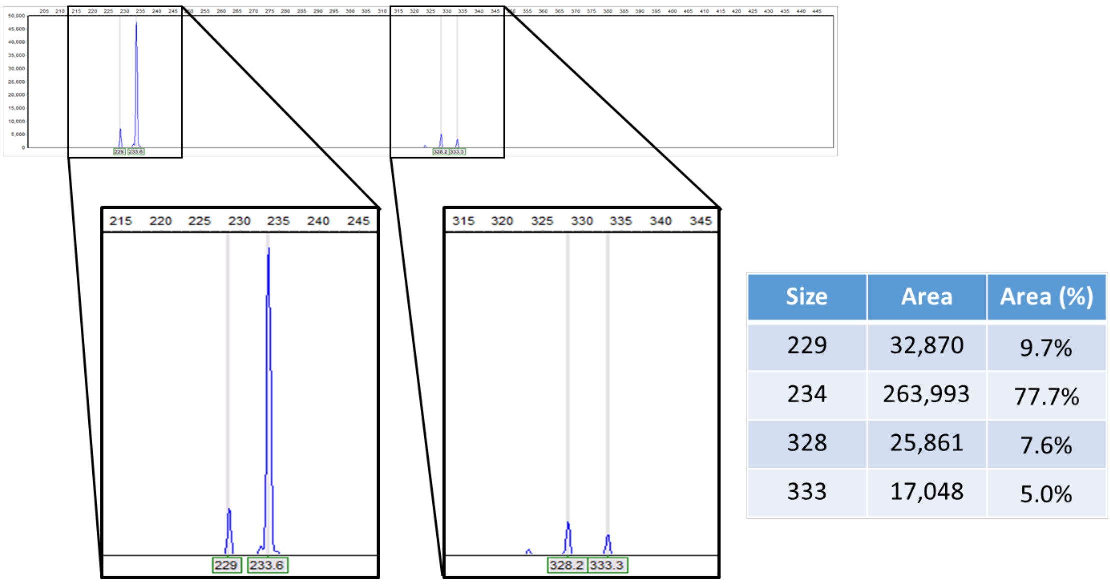

Size	Area	Area (%)
229	32,870	9.7%
234	263,993	77.7%
328	25,861	7.6%
333	17,048	5.0%

a Fragment length analysis of *MLH1* transcript PCR products from Control 1 peripheral blood leukocyte (PBL) cDNA (shown in Figure 4b). 81.9% of the transcript amplicons are of the expected 333bp of wild type transcript sequence. The 328bp product is likely caused by alternate splicing using a cryptic splice donor site in *MLH1* exon 3 (see Supplementary Figure S3a). **b** Fragment length analysis of *MLH1* transcript PCR products from the Case 2 patient PBL cDNA (shown in Figure 4b). 5.0% of the transcript amplicons are of the expected 333bp of wild type transcript sequence. The 328bp product is likely caused by alternate splicing using a cryptic splice donor site in *MLH1* exon 3 (see Supplementary Figure S3a). 77.7% of transcript amplicons are 234bp showing skipping of 99bp of *MLH1* exon 3 caused by the patient's homozygous *MLH1* c.306G>A variant. The 229bp product is likely caused by alternate splicing using the cryptic splice acceptor site in *MLH1* exon 2 (see Supplementary Figure S3b).

Supplementary Figure S5: Sanger sequencing of *MLH1* exon 3 RTB minigene transcript amplicons.

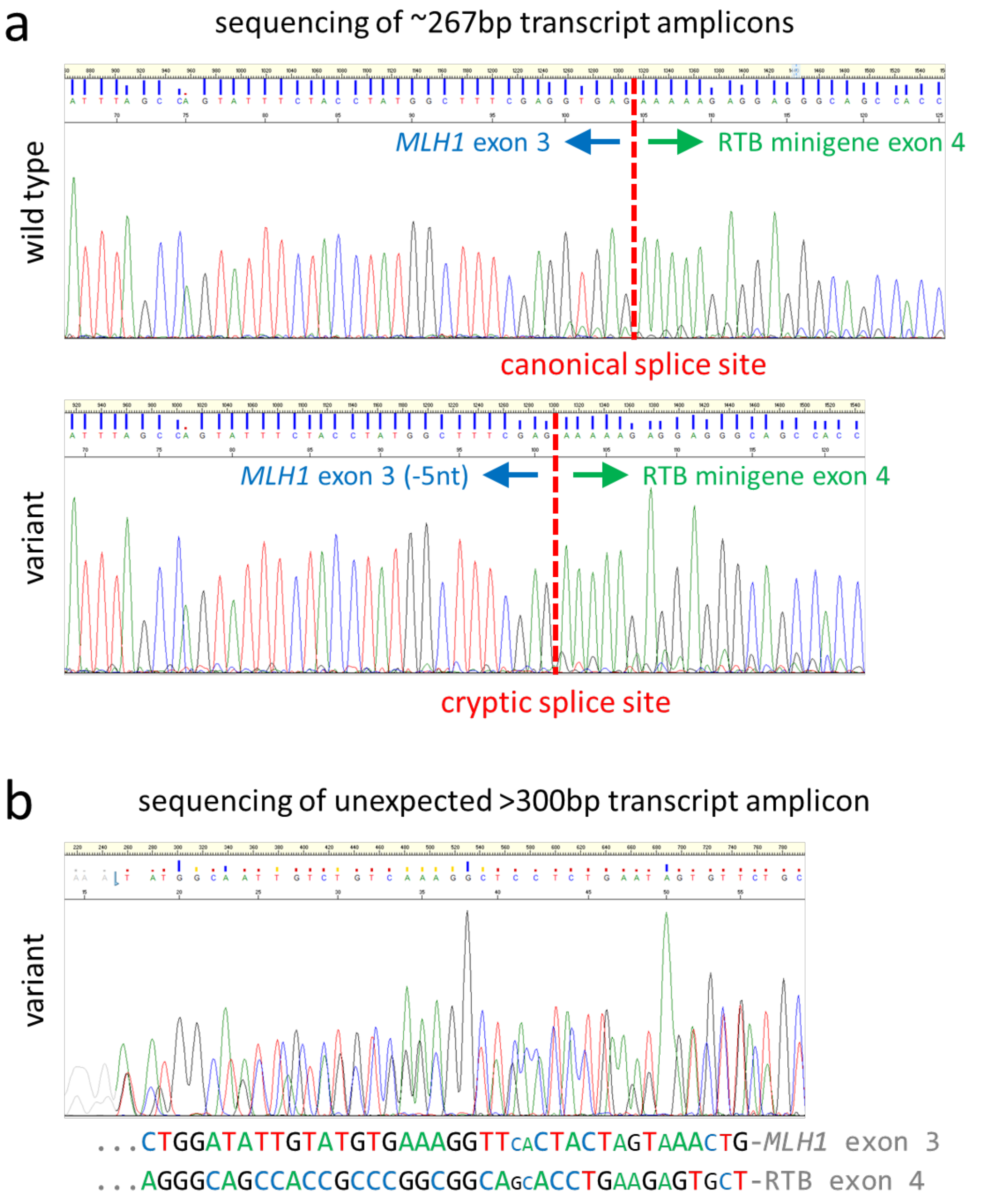

a Sanger sequencing of the ~267bp transcript amplicons from the wild type *MLH1* exon 3 minigene compared to the c.306G>A variant *MLH1* exon 3 minigene (see Figure 4c). The wild type ~267bp amplicon contains the full sequence of *MLH1* exon 3, ending at the natural splice donor site, followed by the RTB minigene exon 4 sequence. However, a weaker sequence signal for the RTB minigene exon 4 starts 5nt upstream from the natural splice site at the same position as a predicted cryptic splice donor site (see Supplementary Figure S3a). The inverse is true for the variant ~267bp amplicon. It contains a shortened sequence of *MLH1* exon 3, ending at the cryptic splice donor site, followed by the RTB minigene exon 4 sequence, and a weaker sequence signal is visible for the full sequence of *MLH1* exon 3. **b** Start of the Sanger sequencing of the unexpected >300bp transcript amplicon from the c.306G>A variant *MLH1* exon 3 minigene (see Figure 4c). The >300bp amplicon appears to consist of two products in a heteroduplex with sequence starting with either *MLH1* exon 3 (transcript amplicons with *MLH1* exon 3) or RTB minigene exon 4 (transcript amplicons skipping *MLH1* exon 3). Note: some bases are in smaller font size to align with the electropherogram trace.

Supplementary Figure S6: Additional assays of constitutional MMR function for Case 2.

a

Sample	Marker		PCR 1	PCR 2	PCR 3	Mean	Threshold
Patient	D2S123	MSIA	0.110	0.096	0.120	**0.109**	0.099
Patient	D17S250	MSIB	0.061	0.061	0.068	0.063	0.106
Patient	D17S791	MSIC	0.141	0.159	0.140	**0.147**	0.145
Control 1	D2S123	MSIA	0.041	0.040	0.036	0.039	0.099
Control 1	D17S250	MSIB	0.051	0.056	0.061	0.056	0.106
Control 1	D17S791	MSIC	0.121	0.083	0.093	0.099	0.145
Control 2	D2S123	MSIA	0.036	0.036	0.037	0.036	0.099
Control 2	D17S250	MSIB	0.047	0.046	0.046	0.046	0.106
Control 2	D17S791	MSIC	0.079	0.108	0.080	0.089	0.145
Control 3	D2S123	MSIA	0.029	0.027	0.028	0.028	0.099
Control 3	D17S250	MSIB	0.078	0.076	0.101	0.085	0.106
Control 3	D17S791	MSIC	0.085	0.083	0.080	0.083	0.145

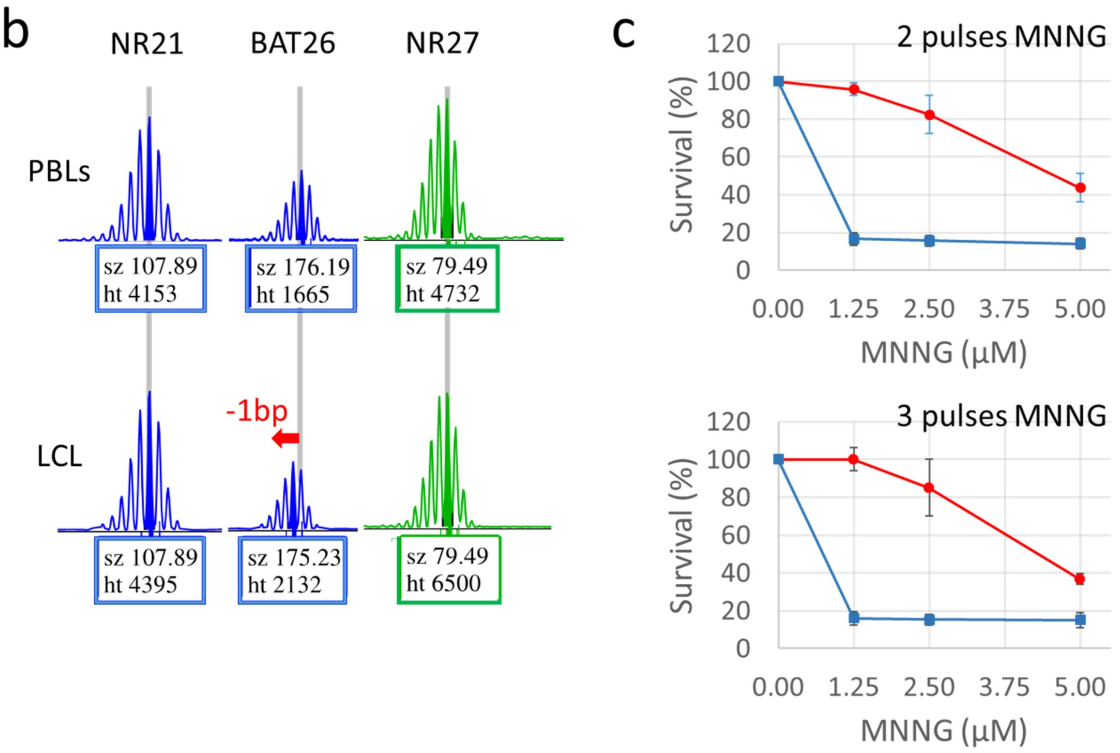

a gMSI analysis of Case 2 patient and control peripheral blood leukocyte (PBL) DNA using the method of Ingham et al. (2013; PMID: 23483711). Values represent the peak height ratio. The

patient has 2/3 markers with mean peak height ratios greater than internally validated thresholds (the mean of 40 controls + 3 standard deviations, as described by Gallon et al., 2019; PMID: 30740824), showing presence of gMSI and indicating CMMRD. **b** ex vivo MSI (evMSI) analysis of Case 2 patient PBL and lymphoblastoid cell line (LCL) DNA using the method of Bodo et al. (2015; PMID: 26116798). The electropherograms show the sizes of fluorescent amplicons for NR21, BAT26, and NR27 microsatellites. The length of the predominant allele in base pairs (bp) and the fluorescence intensity are indicated in the box below each profile. A 1bp deletion (red arrow) was found in BAT26 in the patient LCL, showing presence of evMSI and indicating CMMRD. The LCL was cultured for 392 days. Control cultures up to 304 days have not shown evidence of evMSI (Bodo et al., 2015). **c** Methylation tolerance analysis of Case 2 patient and control LCLs using the method of Bodo et al. (2015). Control LCLs were derived from individuals without germline pathogenic variants in the MMR genes. The survival of the patient LCLs (red line) and control LCLs (blue line) following 2 or 3 pulses of increasing concentrations of the methylating agent 1-methyl-3-nitro-1-nitrosoguanidine (MNNG) is shown. Bodo et al. defined thresholds using LCLs from CMMRD patients and from controls, including 40 patients with Lynch syndrome and 12 patients with tumour predisposition syndromes caused by pathogenic variants in other, non-MMR genes (such as familial adenomatous polyposis or neurofibromatosis type 1). After 2 rounds of 2.5uM MNNG, LCLs from all 14 CMMRD patients displayed a cell survival rate above 60%, whereas 51/52 LCLs from controls displayed a cell survival rate lower than 40%. Here, the patient LCLs have >60% survival after 2 or 3 pulses of 2.5µM MNNG, showing methylation tolerance and indicating CMMRD.

Supplementary Figure S7: Schematic of the RTB minigene construct containing *MLH1* exon 3.

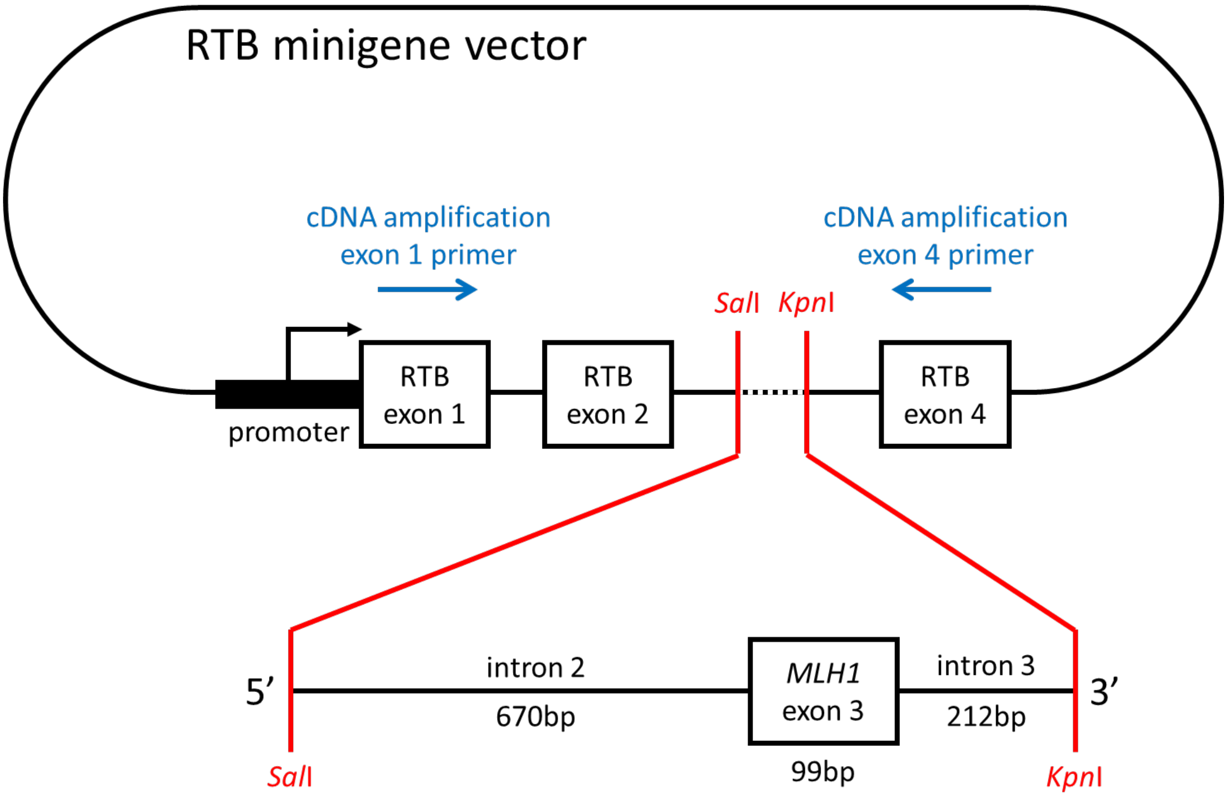

Two RTB minigene constructs (Ryan & Cooper, 1996; PMID: 8754799) were generated containing either wild type *MLH1* exon 3 or *MLH1* exon 3 with the c.306G>A variant.

Supplementary Table S1: Genotypes and phenotypes of individuals carrying the *MSH6* c.3226C>T p.(Arg1076Cys) variant.

Reference	Published Patient ID	Genotype	Variant 1	Variant 2	Clinical Presentation	Phenotype Summary
Plaschke et al. 2006 PMID: 16418736	HD3	*MSH6* comp. het.	c.3226C>T p.(Arg1076Cys) missense	c.3991C>T p.(Arg1331*) truncating	CRC (19 years, lost MSH6 expression including normal tissue, MSI-high), EC (24 years, lost MSH6 expression including normal tissue), CALMs.	CMMRD-like.
	NA	*MSH6* het.	c.3226C>T p.(Arg1076Cys) missense	NA	No cancer (57 years), mother of patient HD3.	No cancer.
Okkels et al. 2006 PMID: 16525781	IV2	*MSH6* comp. het.	c.3226C>T p.(Arg1076Cys) missense	c.1836C>A p.(Ser612*) truncating	CRC (18 years, lost MSH6 expression), 19 GIPs (18 years).	CMMRD-like.
	III3	*MSH6* het.	c.3226C>T p.(Arg1076Cys) missense	NA	No cancer (56 years), father of patient IV2.	No cancer.
	III2	*MSH6* het.	c.3226C>T p.(Arg1076Cys) missense	NA	No cancer (50 years), paternal aunt of patient IV2.	No cancer.
Rahner et al. 2008 PMID: 18409202	NA	*MSH6* comp. het.	c.3226C>T p.(Arg1076Cys) missense	c.1806_1809delAAAG p.(Glu604Leufs*5) truncating	Systemic lupus erythematosus (16 years), 4 synchronous CRCs (17 years, lost MSH6 expression including normal tissue, MSI-high), 3 GIPs (17 years), vitiligo.	CMMRD-like.
Nilbert et al. 2009 PMID: 18566915	NA	*MSH6* het.	c.3226C>T p.(Arg1076Cys) missense	NA	2 families reported in the Danish HNPCC-Register, no further data.	LS-like.
Schofield et al. 2009 PMID: 19072991	98	*MSH6* het.	c.3226C>T p.(Arg1076Cys) missense	NA	GC (undisclosed age), CRC (55 years, subclonally-lost MSH6 expression, MSI-high). Fulfils Amsterdam criteria (sibling CRC, aunt OC, cousin EC).	LS-like.
Jasperson et al. 2011 PMID: 21039432	III-1	*MSH6* comp. het.	c.3226C>T p.(Arg1076Cys) missense	c.1421_1422dupTG p.(Gln475fs) truncating	6 GIP (21 years, lost MSH6 expression in neoplastic cells only, MSI-low result for the one adenoma analysed), CALMs.	CMMRD-like.
	III-2	*MSH6* comp. het.	c.3226C>T p.(Arg1076Cys) missense	c.1421_1422dupTG p.(Gln475fs) truncating	>20 GIP (23 years, lost MSH6 expression in neoplastic cells only), CALMs, skin-fold freckling.	CMMRD-like.
	III-3	*MSH6* het.	c.3226C>T p.(Arg1076Cys) missense	NA	No cancer (undisclosed age), half-sibling of III-1 and III-2.	No cancer.
	II-5	*MSH6* het.	c.3226C>T p.(Arg1076Cys) missense	NA	No cancer (undisclosed age), mother of III-1 and III-2.	No cancer.
Limburg et al. 2011 PMID: 21056691	27	*MSH6* het.	c.3226C>T p.(Arg1076Cys) missense	NA	CRC (46 years, lost MSH6 expression).	LS-like.
Klarskov et al. 2011 PMID: 21836479	356-8	*MSH6* comp. het.	c.3226C>T p.(Arg1076Cys) missense	c.1835C>A p.(Ser612*) truncating	CRC (43 years, lost MSH6 expression, MSI-high).	LS-like.
	356-10	*MSH6* comp. het.	c.3226C>T p.(Arg1076Cys) missense	c.1835C>A p.(Ser612*) truncating	CRC (18 years, lost MSH6 expression, MSI-high).	CMMRD-like.
Lagerstedt-Robinson et al. 2016 PMID: 27601186	NA	*MSH6* het.	c.3226C>T p.(Arg1076Cys) missense	NA	1 family reported from LS famiies idenitified from Swedish hospital laboratories/oncogenetic clinics. No further data.	LS-like.
Local observations, Centre for Human Genetics, University Hospital Leuven, Belgium, and Institute of Human Genetics, Medical University Innsbruck, Austria	NA	*MSH6* het.	c.3226C>T p.(Arg1076Cys) missense	NA	No cancer (undisclosed age), variant found incidentally.	No cancer.
	NA	*MSH6* het.	c.3226C>T p.(Arg1076Cys) missense	NA	BrC (undisclosed age).	Non-LS spectrum cancer.
	NA	*MSH6* het.	c.3226C>T p.(Arg1076Cys) missense	NA	CRC (undisclosed age; retained MSH6 expression, MSS, no second hit or loss of heterozygosity of MSH6).	LS-spectrum cancer but MMR proficient.
	NA	*MSH6* het.	c.3226C>T p.(Arg1076Cys) missense	NA	No cancer (55 years), no FH of LS-associated cancers, variant found incidentally.	No cancer.

comp. het. - compound heterozygous; het. - heterozygous; BrC - breast cancer; CRC - colorectal cancer; DC - duodenal cancer; EC - endometrial cancer; GBM - glioblastoma; GC - gastric cancer; GIP - gastrointestinal polyps; OC- ovarian cancer; PrC - prostate cancer; CALM - cafe au lait macule; CMMRD - constitutional mismatch repair deficiency; FH - family history; HNPCC - hereditary non-polyposis colorectal cancer; LS - Lynch syndrome; MSI - microsatellite instability; MSS - microsatellite stable.

Supplementary Table S2: Somatic *POLE* variants detected in the Case 1 glioblastoma.

cDNA change	Variant allele frequency	Protein change	Domain	Conservation	AlphaMissense score	Previously reported	Notes
c.1288G>T	0.347	p.(Ala430Ser)	Exonuclease domain	Highly conserved in higher eukaryotes.	0.118 (benign)	Reported once in ClinVar as a VUS from a suspected hereditary CRC case. Otherwise, not found in the literature, GnomAD, or COSMIC database at the time of this study.	The equivalent residue in yeast is a serine.
c.2385G>T	0.316	p.(Lys795Asn)	Polymerase domain	Highly conserved.	0.538 (ambiguous)	No - not found in the literature, GnomAD, or COSMIC database at the time of this study.	The AlphaMissense score is close to the threshold (0.564) for classification as pathogenic.
c.3974G>T	0.323	p.(Ser1325Ile)		Highly conserved.	0.416 (ambiguous)	No - not found in the literature, GnomAD, or COSMIC database at the time of this study.	
c.5659G>T	0.317	p.(Val1887Leu)		Conserved.	0.261 (benign)	No - not found in the literature, GnomAD, or COSMIC database at the time of this study.	

CRC - colorectal cancer; VUS - variant of uncertain significance.

Supplementary Table S3: Variants detected in reported cases of late onset CMMRD.

Gene	Transcript ID	cDNA change	SpliceAI delta score	phyloP nucleotide conservation score	Protein change	Coding effect	AlphaMissense score	REVEL score
MLH1	NM_000249.4	c.306G>A	Donor Loss: 0.88†	5.81	p.[(Lys70_Glu102del,Glu102=)]	loss of 33 AA, silent	NA, NA	NA, NA
MSH2	NM_000251.3	c.188T>A	NA	7.39	p.(Val63Glu)	missense	0.987	0.923
MSH6	NM_000197.3	c.3226C>T	NA	3.09	p.(Arg1076Cys)	missense	0.453	0.849
MSH6	NM_000197.3	c.1421_1422dupTG	NA	NA	p.(Gln475Glyfs*7)	truncating	NA	NA
PMS2	NM_000535.7	c.2531C>A	NA	9.87	p.(Pro844His)	missense	0.982	0.808
PMS2	NM_000535.7	c.2002A>G	Donor Loss: 0.49†<br>Donor Gain: 0.97†	7.29	p.[Ile668*,Ile668Val)]	truncating, missense	NA, 0.246	NA, 0.133
PMS2	NM_000535.7	c.137G>T	NA	7.43	p.(Ser46Ile)	missense	0.954	0.939
PMS2	NM_000535.7	c.1261C>T	NA	1.2	p.(Arg421*)	truncating	NA	NA

AA - amino acid. † The variant causes a "leaky" splice effect.

SpliceAI (Jaganathan et al. 2019, PMID: 30661751) thresholds:

- delta score <0.1 = splice effect highly unlikely
- delta score 0.1-0.2 = splice effect unlikely
- delta score 0.2-0.5 = splice effect possible
- delta score 0.5-0.8 = splice effect likely
- delta score >0.8 = splice effect almost certain

REVEL (Ioannidis et al. 2016, PMID: 27666373) does not define specific thresholds, but the following can be used as a guide:

- REVEL score >0.50 for the classification of disease causing variants = 75.4% sensitivity and 89.1% specificity
- REVEL score >0.75 for the classification of disease causing variants = 52.1% sensitivity and 96.7% specificity

AlphaMissense (Cheng et al. 2023, PMID: 37733863) thresholds:

- AlphaMissense score <0.34 = benign
- AlphaMissense score 0.34-0.564 = ambiguous
- AlphaMissense score >0.564 = pathogenic
