# Supplementary material for: Constitutional mismatch repair deficiency mimicking Lynch syndrome is associated with hypomorphic mismatch repair gene variants
Source: NPJ Precis Oncol. 2024 May 24;8:119. doi: 10.1038/s41698-024-00603-z (PMC11126593; doi:10.1038/s41698-024-00603-z)
Supplement: Supplementary file 2 — Reporting summary [file 41698_2024_603_MOESM2_ESM.pdf]

nature portfolio

Corresponding author(s): Dr Richard Gallon

Last updated by author(s): Feb 9, 2024

Reporting Summary

Nature Portfolio wishes to improve the reproducibility of the work that we publish. This form provides structure for consistency and transparency in reporting. For further information on Nature Portfolio policies, see our Editorial Policies and the Editorial Policy Checklist.

Statistics

For all statistical analyses, confirm that the following items are present in the figure legend, table legend, main text, or Methods section.

n/a	Confirmed	
☐	☒	The exact sample size (*n*) for each experimental group/condition, given as a discrete number and unit of measurement
☒	☐	A statement on whether measurements were taken from distinct samples or whether the same sample was measured repeatedly
☐	☒	The statistical test(s) used AND whether they are one- or two-sided<br>*Only common tests should be described solely by name; describe more complex techniques in the Methods section.*
☒	☐	A description of all covariates tested
☒	☐	A description of any assumptions or corrections, such as tests of normality and adjustment for multiple comparisons
☒	☐	A full description of the statistical parameters including central tendency (e.g. means) or other basic estimates (e.g. regression coefficient) AND variation (e.g. standard deviation) or associated estimates of uncertainty (e.g. confidence intervals)
☐	☒	For null hypothesis testing, the test statistic (e.g. *F*, *t*, *r*) with confidence intervals, effect sizes, degrees of freedom and *P* value noted<br>*Give P values as exact values whenever suitable.*
☒	☐	For Bayesian analysis, information on the choice of priors and Markov chain Monte Carlo settings
☒	☐	For hierarchical and complex designs, identification of the appropriate level for tests and full reporting of outcomes
☒	☐	Estimates of effect sizes (e.g. Cohen's *d*, Pearson's *r*), indicating how they were calculated

Our web collection on statistics for biologists contains articles on many of the points above.

Software and code

Policy information about availability of computer code

Data collection	Nucleotide conservation scores generated by phyloP100way were obtained from Varsome (https://varsome.com/). Prediction of splice site effects of nucleotide variants used spliceAI scores obtained from SpliceAI Lookup with default settings (https://spliceailookup.broadinstitute.org/). Prediction of functional effects of missense variants used REVEL scores and AlphaMissense scores obtained from https://zenodo.org/records/7072866 and https://zenodo.org/records/8360242, respectively. cMSI analysis used custom software written in R (https://cran.r-project.org/) according to Gallon et al. 2023 (PMID: 36586540). gMSI analysis used the PeakHeights software (http://dna.leeds.ac.uk/peakheights/). Fragment length analysis used GeneMapper 4.1 software (Applied Biosystems, Vienna, Austria). Sanger sequencing data analysis used the SeqNext version 26 software (JSI medical systems, Ettenheim, Germany) and the Sequence Scanner Software v1.0 (Applied Biosystems).
Data analysis	Data analysis used R version 4.2.2 (https://cran.r-project.org/).

For manuscripts utilizing custom algorithms or software that are central to the research but not yet described in published literature, software must be made available to editors and reviewers. We strongly encourage code deposition in a community repository (e.g. GitHub). See the Nature Portfolio guidelines for submitting code & software for further information.

Data

Policy information about availability of data

All manuscripts must include a data availability statement. This statement should provide the following information, where applicable:

- Accession codes, unique identifiers, or web links for publicly available datasets
- A description of any restrictions on data availability
- For clinical datasets or third party data, please ensure that the statement adheres to our policy

Data sharing is not applicable to this article as no datasets were generated or analysed during the current study.

Research involving human participants, their data, or biological material

Policy information about studies with human participants or human data. See also policy information about sex, gender (identity/presentation), and sexual orientation and race, ethnicity and racism.

Reporting on sex and gender	Case patient sex is appropriately reported as it is relevant to the clinical presentation, in particular the diagnoses of cancers of the female reproductive system.
Reporting on race, ethnicity, or other socially relevant groupings	Ethnicity is only described in the appropriate context of the population frequency of genetic variants identified in the two case patients described. Controlling for variables relating to race, ethnicity, or other social grouping was not otherwise relevant for this study.
Population characteristics	NA
Recruitment	NA
Ethics oversight	The case reports presented here have followed CARE guidelines where possible (https://www.care-statement.org/). Both patients consented to publication of de-identified information relevant to the clinical presentation and management of their disease.

Note that full information on the approval of the study protocol must also be provided in the manuscript.

Field-specific reporting

Please select the one below that is the best fit for your research. If you are not sure, read the appropriate sections before making your selection.

☒ Life sciences ☐ Behavioural & social sciences ☐ Ecological, evolutionary & environmental sciences

For a reference copy of the document with all sections, see nature.com/documents/nr-reporting-summary-flat.pdf

Life sciences study design

All studies must disclose on these points even when the disclosure is negative.

Sample size	NA
Data exclusions	NA
Replication	NA
Randomization	NA
Blinding	NA

Reporting for specific materials, systems and methods

We require information from authors about some types of materials, experimental systems and methods used in many studies. Here, indicate whether each material, system or method listed is relevant to your study. If you are not sure if a list item applies to your research, read the appropriate section before selecting a response.

Materials & experimental systems

n/a	Involved in the study
☒	☐ Antibodies
☒	☐ Eukaryotic cell lines
☒	☐ Palaeontology and archaeology
☒	☐ Animals and other organisms
☒	☐ Clinical data
☒	☐ Dual use research of concern
☒	☐ Plants

Methods

n/a	Involved in the study
☒	☐ ChIP-seq
☒	☐ Flow cytometry
☒	☐ MRI-based neuroimaging

Plants

Seed stocks	NA
Novel plant genotypes	NA
Authentication	NA
